# High-Throughput Screening for GPR119 Modulators Identifies a Novel Compound with Anti-Diabetic Efficacy in *db/db* Mice

**DOI:** 10.1371/journal.pone.0063861

**Published:** 2013-05-21

**Authors:** Meng Zhang, Yang Feng, Jia Wang, Jianwei Zhao, Ting Li, Min He, Dehua Yang, Olivier Nosjean, Jean Boutin, Pierre Renard, Ming-Wei Wang

**Affiliations:** 1 The National Center for Drug Screening, State Key Laboratory of Drug Research, Shanghai Institute of Materia Medica, Chinese Academy of Sciences, Shanghai, China; 2 Chinese National Compound Library, Shanghai, China; 3 Les Laboratoires Servier, Neuilly-sur-Seine, France; University of Hong Kong, China

## Abstract

G protein-coupled receptor 119 (GPR119) is highly expressed in pancreatic β cells and enteroendocrine cells. It is involved in glucose-stimulated insulin secretion and glucagon-like peptide-1 (GLP-1) release, thereby representing a promising target for the treatment of type 2 diabetes. Although a number of GPR119 agonists were developed, no positive allosteric modulator (PAM) to this receptor has been reported. Here we describe a high-throughput assay for screening GPR119 PAMs and agonists simultaneously. Following screening of a small molecule compound library containing 312,000 synthetic and natural product-derived samples, one potent GPR119 agonist with novel chemical structure, MW1219, was identified. Exposure of MIN6 and GLUTag cells to MW1219 enhanced glucose-stimulated insulin secretion and GLP-1 release; once-daily oral dosing of MW1219 for 6 weeks in diabetic *db/db* mice reduced hemoglobin A1c (HbA1c) and improved plasma glucose, insulin and GLP-1 levels; it also increased glucose tolerance. The results demonstrate that MW1219 is capable of effectively controlling blood glucose level and may have the potential to be developed as a new class of anti-diabetic agents.

## Introduction

Type 2 diabetes mellitus (T2DM), characteristic of defects in both insulin secretion and sensitivity [1], [2], is an increasing threat to human health. Due to the multiplicity of pathologies and the complexity of control mechanism of human body, there exist many therapeutics for T2DM presently [3].

In recent years, compounds that enhance incretin activities have been of particular interest to pharmaceutical companies. Incretin-based therapies are also becoming popular which use either GLP-1 mimetics or DPP-4 inhibitors [4]. However, each has shown its limitations clinically. For example, the efficacy of DPP-4 inhibitors is modest because their action is dependent upon endogenous GLP-1 while GLP-1 mimetics require frequent injections [5]. Therefore, the strategy to identify orally active agents capable of stimulating GLP-1 release remains attractive [6].

GPR119 is a member of class A G protein-coupled receptor (GPCR) family. It is highly expressed in pancreatic β-cells and intestinal endocrine cells [7]. Additionally, GPR119 mRNA is known to be significantly elevated in the islets of obese *db/db* mice compared with that of normal [8]. Upon activation by endogenous ligand, oleoylethanolamide (OEA), the resultant accumulation of intracellular cAMP via adenylate cyclase activation enhances the effect of glucose-stimulated insulin secretion (GSIS) and GLP-1 release, thus GPR119 represents a promising target for the treatment of type 2 diabetes [7], [9], [10] and a potential product development attraction to many drug makers. Based on the expression profile and biological actions of GPR119, a number of small molecule GPR119 agonists have been reported [11] but efforts to discover positive allosteric modulators (PAMs) met with difficulties. Allosteric modulators bind to sites different from endogenous ligands. Due to the fact that they are able to provide receptor specificity and selectivity, allosteric modulation has gained much traction as a means to overcome the limitations of many orthosteric ligands [12].

In this paper, we studied the allosteric activity between OEA and the first small molecule GPR119 agonist, AR-231453 [13]. A high-throughput screening (HTS) assay was developed and applied to search for GPR119 PAMs and agonists. A novel GPR119 agonist (MW1219) was subsequently identified and characterized both *in vitro* and *in vivo* using a variety of bioassays as well as diabetic *db/db* mice.

## Materials and Methods

### Cell Culture

HEK293 cells stably transfected with a human GPR119 vector and a cAMP response element-driven luciferase reporter plasmid (HEK293-hGPR119 cells) and HEK293 cells transfected only with a cAMP response element-driven luciferase reporter plasmid (control HEK293 cells) were cultured in Dulbecco’s modified Eagle medium (DMEM) (Invitrogen, Carlsbad, CA, USA) supplemented with 10% fetal bovine serum (FBS), 4.5 g/l glucose, 100 units/ml penicillin and 100 µg/ml streptomycin at 37°C in 5% CO_2_. MIN6 cells were maintained in DMEM containing 15% FBS, 4.5 g/l glucose, 100 units/ml penicillin and 100 µg/ml streptomycin at 37°C in 5% CO_2_. GLUTag cells were maintained in DMEM containing 10% FBS, 1 g/l glucose, 100 units/ml penicillin and 100 µg/ml streptomycin at 37°C in 5% CO_2_.

### Reporter Gene Assay

HEK293-hGPR119 cells or control HEK293 cells were seeded onto 384-well plates with a density of 16,000 cells per well. At the time of assaying, AR-231453 or test compounds dissolved in DMSO were added. After 42 h of incubation at 37°C and 5% CO_2_ in a cell culture incubator, cells were lysed and quantified for luciferase activity using the Steady-Glo luciferase assay system (Promega, Madison, WI, USA) according to manufacturer’s protocol. For allosteric activity studies, test compounds were 5-fold serially diluted and added to AR-231453 or OEA concentration response reaction. Luciferase signals were determined with EnVision (PerkinElmer, Boston, MA, USA).

### cAMP Accumulation Assay

cAMP accumulation was measured using HTRF-cAMP dynamic kit (Cisbio International, Gif sur Yvette Cedex, France) according to manufacturer's instructions. Briefly, HEK293-hGPR119 cells were suspended in assay buffer (DMEM, 1 mM 3-isobutyl-1-methylxanthine) and transferred to 384-well microplates (Greiner Bio-One, Frickenhausen, Germany) at a density of 16,000 cells/well. Plates were incubated for 30 min at 37°C before adding test compounds. After treatment for 30 min at 37°C, the reactions were stopped by addition of lysis buffer containing HTRF reagents. Plates were then incubated for 60 min at room temperature, and time-resolved FRET signals were measured after excitation at 320 nm. Both the emission signal from the europium cryptate-labeled anti-cAMP antibody (620 nm) and the FRET signal resulting from the labeled cAMP-d2 (665 nm) were detected by EnVision (PerkinElmer).

### Insulin Secretion Assay

Insulin-secreting MIN6 cells were plated in 96-well plates (20,000 cells per well) for 2 days. On the day of experiment, culture medium was aspirated and cells were washed twice with KRBH buffer. Cells were then placed at 37°C for 30 min in KRBH containing 2.8 mM glucose. Test compounds were dissolved in either 2.8 or 16.8 mM glucose medium and added to cells for 1 h. The supernatants were collected by centrifugation at 800×g for insulin measurements. Insulin content in the supernatant was determined using an insulin ELISA kit from Linco Research Laboratory (Billerica, MA, USA).

### GLP-1 Release Assay

GLUTag cells were plated in 24-well plates on day 1 in low glucose DMEM supplemented with 10% FBS. Culture medium was replaced with DMEM (2.8 mM glucose) supplemented with 10% FBS 24 h before analysis of GLP-1 release. On the day of experiment, cells were washed twice with PBS and incubated with different compounds at desired concentrations in serum-free DMEM with 2.8 mM or 16.8 mM glucose for 1 h at 37°C and 5% CO_2_. The supernatants were collected by centrifugation for 3 min. GLP-1 in the supernatant was detected by a GLP-1 ELISA kit (Linco Research Laboratory)**.**

### Small Interfering RNA Transfection

MIN6 and GLUTag cells were cultured as described above. siRNA targeting murine GPR119 coding sequences (Forward: 5′-CUAUGCUGCUAUCAAUCUATT-3′, Reverse: 5′-UAGAUUGAUAGCAGCAUAGTT-3′) was purchased from GenePharma company (Shanghai, China). Transfection was performed using siRNA and Lipofectamine 2000 (Invitrogen, Burlington, ON, Canada) as instructed by the manufacturer. After 36 h, knockdown efficiency of siRNA was quantified by real-time RT-PCR, and cells were used for secretion experiments.

### Animal Experiments

Animal experimentation was conducted in accordance with the regulations adopted by the Animal Care and Use Committee, Shanghai Institute of Materia Medica, Chinese Academy of Sciences (approval number: SIMM-2012-07-WMW-04). Male C57BL/KsJ *db/db* mice (Model Animal Research Center of Nanjing University, Nanjing, China) were housed in a temperature-controlled room (22±2°C), with a light/dark cycle of 12 h. At 6 weeks of age, mice were randomly assigned to chronic treatments. The experimental groups and respective doses of compounds were as follows: (1) 0.5% w/v sodium carboxyl methyl cellulose (CMC), (2) 10 mg/kg AR-231453, (3) 1 mg/kg sitagliptin (Beijing Huikang Boyuan Chemical Technology Co., Ltd., Beijing, China), (4) 10 mg/kg AR-231453 plus 1 mg/kg sitagliptin, (5) 100 mg/kg MW1219, and (6) 100 mg/kg MW1219 plus 1 mg/kg sitagliptin. Each regimen was administered once daily by oral gavaging for 6 weeks. Body weight and food intake were recorded every other day and fasting glucose levels measured every week. Oral glucose tolerance test was performed in overnight-fasting mice every other week. After six weeks of treatment, HbA1c were quantified, plasma insulin and GLP-1 contents were measured using respective ELISA kit. For oral glucose tolerance test, overnight fasting mice (n = 6/treatment) were given compounds orally at desired doses and after 30 min, an oral glucose bonus (3 g/kg) was delivered. Plasma glucose levels were determined at desired time points over a 2-h period using blood collected from the tail vein. For circulating GLP-1 analysis, compounds were administered orally to fasting animals followed by an oral glucose bolus (3 g/kg) 30 min later. Blood was drawn 2 min thereafter in Eppendorf tubes containing EDTA and a DPP-4 inhibitor. Plasma samples were obtained via centrifugation and assayed for active GLP-1 by the ELISA kit. Blood was collected again after 20 min to determine insulin levels.

### Statistical Analysis

Results are presented as means ± SEM. Differences between groups were analyzed by One-Way ANOVA. All statistical analysis was performed using Prism statistical methods (GraphPad, San Diego, CA, USA).

## Results

### Assay Development for Identifying GPR119 Modulators

**Figure 1:**
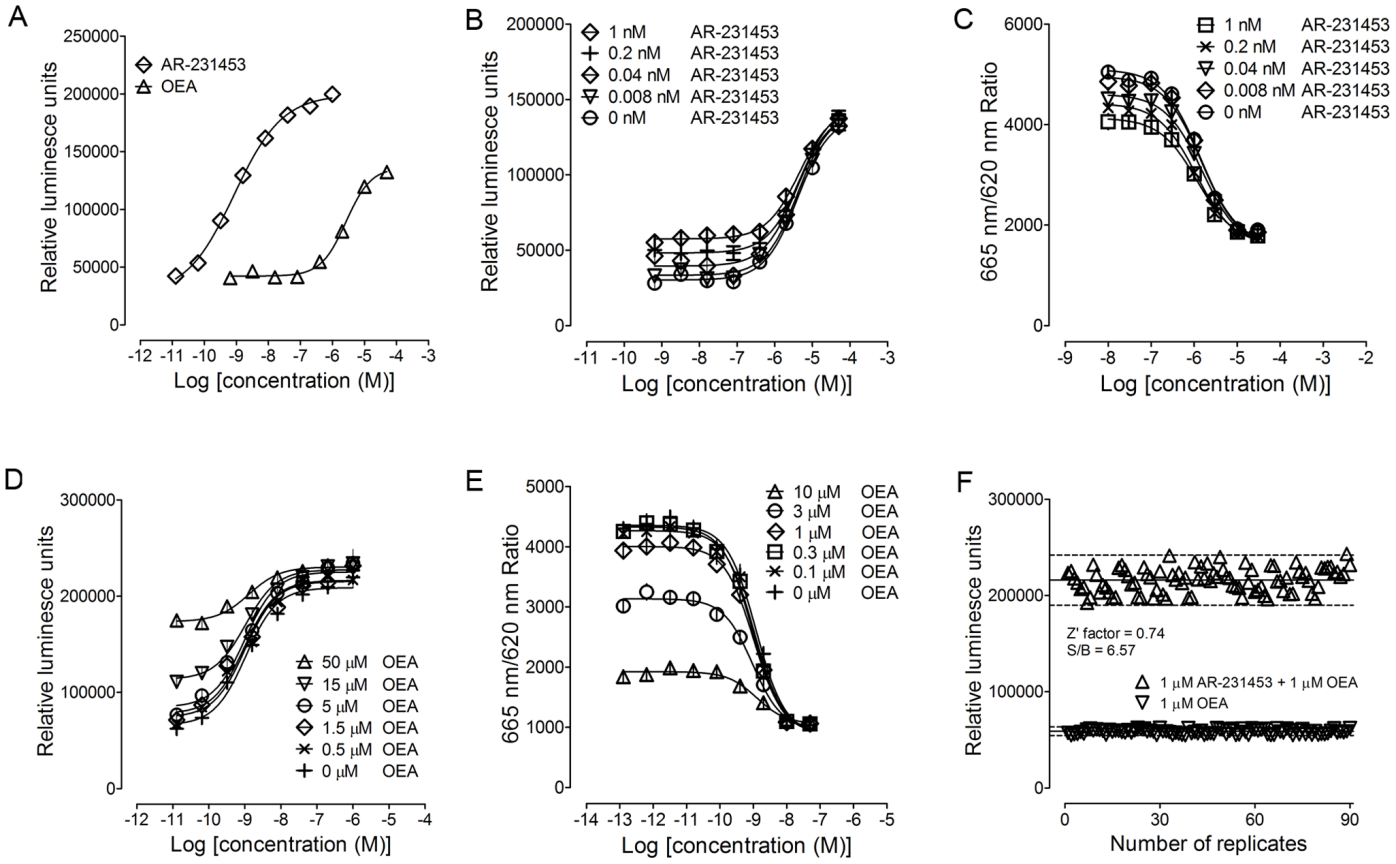
Biological activities of AR-231453 and OEA ***in vitro*** **and development of a high-throughput screening assay for GPR119 modulators.** Agonist activities of AR-231453 and OEA were measured with a reporter gene assay (A), in which allosteric properties of both were studied in the presence of different concentrations of (B) AR-231453 *vs*. OEA or (D) OEA *vs*. AR-231453. The experiments were repeated in a cAMP accumulation assay (C and E). (F) Validation of the high-throughput screening assay. All data points were representatives of three independent experiments, determined in triplicate.

Both AR-231453 and OEA could stimulate luciferase expression in HEK293-hGPR119 cells with EC_50_ values measured at 1.05±0.11 nM and 2.78±0.18 µM, respectively (Figure 1A), consistent with those reported in the literature [13], [14]. It is thus established that this cell line stably transfected with human GPR119 is suitable for screening purposes.

Although OEA and AR-231453 behave as agonists in GPR119-related bioassays, given their difference in chemical structures, we investigated the question whether their effects are mediated through the same binding site on GPR119. A series of compound combination studies using both reporter gene and cAMP accumulation assays were carried out. It was found that AR-231453 was able to increase basal luciferase and cAMP responses elicited by OEA without affecting its potency (Figures 1B and 1C). EC_50_ values are shown in Table S1. Such an increase of basal signal is due to the activity of AR-231453 on GPR119 alone. The lack of a shift in potency of OEA suggests that AR-231453 and OEA either bind to the same site or their binding areas are somehow overlapped. Similarly, addition of OEA to AR-231453 also increased basal luciferase and cAMP levels compared to that induced by AR-231453 alone; again, it did not alter the potency of AR-231453 (Figures 1D and 1E, Table S2).

A number of agonists for GPR119 have been discovered to date, but none of them are PAM. To identify compounds with PAM or agonist activities for GPR119, we optimized the luciferase assay to meet high-throughput screening requirements. It exhibited high signal-to-background ratio and Z′ factor (6.57 and 0.74, respectively) (Figure 1F) [15]. Test compounds obtained from the National Center for Drug Screening and a certain concentration of OEA with 15% efficacy on GPR119 (1 µM) were applied to stimulate HEK293-hGPR119 cells simultaneously. Theoretically, compounds with PAM activity or showing agonist effect on GPR119 (efficacy above 15%) could be selected following HTS campaigns.

### Identification of a Novel GPR119 Agonist by HTS

**Figure 2:**
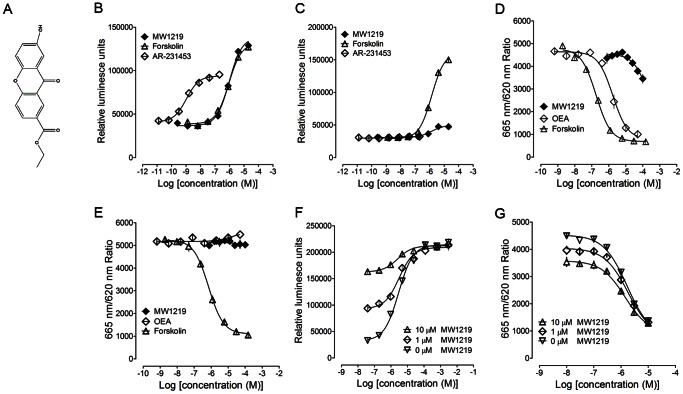
Identification and characterization of a novel GPR119 agonist. (A) Chemical structure of compound MW1219. Agonist activity of MW1219 was studied in HEK293-hGPR119 cells (B) and control HEK293 cells (C) employed in a reporter gene assay system, respectively. The effect of MW1219 was confirmed in a cAMP accumulation assay using HEK293-hGPR119 cells and control HEK293 cells (D and E). Allosteric activities of MW1219 for OEA were measured in both reporter gene and cAMP assays (F and G). All data points were representatives of three independent experiments, determined in triplicate.

A total of 312,000 synthetic and natural compounds were screened against HEK293-hGPR119 cells. Through primary screening and subsequent confirmation studies, a synthetic compound, MW1219 (Figure 2A), was found to invoke luciferase reaction through GPR119 in a concentration-dependent manner with an EC_50_ of 0.96±0.08 µM (Figure 2B). To further study whether the observed cellular responses are receptor-mediated, the effect of MW1219 was measured in control HEK293 cells. In the absence of GPR119, it did not show any activity on luciferase expression thereby demonstrating its specificity for GPR119. Compared to forskolin, efficacy of MW1219 was much lower (10%) which may reflect normal fluctuation of the assay (Figure 2C).

Because GPR119 is mainly coupled to Gα_s_ pathway, the ability of MW1219 to activate this signal transduction route was then explored in a Gα_s_-coupled cAMP accumulation assay. MW1219 only stimulated the increase of cAMP in HEK-hGPR119 cells and had no influence in control HEK239 cells (Figures 2D and 2E). In this system, the potency displayed by MW1219 was different from that seen in the reporter gene assay. An efficacy achieved with 50 µM MW1219 was about 40% of that elicited by forskolin, whereas it could induce the same level of efficacy as forskolin in the reporter gene assay.

To determine whether MW1219 could allosterically modulate the activity of OEA, 10 µM, 1 µM and 0 µM MW1219 were added to various OEA concentrations. The EC_50_ of OEA did not change significantly, suggesting that MW1219 is not PAM for OEA (Figures 2F and 2G, Table S3).

Based on the results presented above, we conclude that MW1219 is an agonist for GPR119 which is capable of activating Gα_s_-coupled signal pathways.

### MW1219 Stimulates Insulin and GLP-1 Secretion in vitro

**Figure 3:**
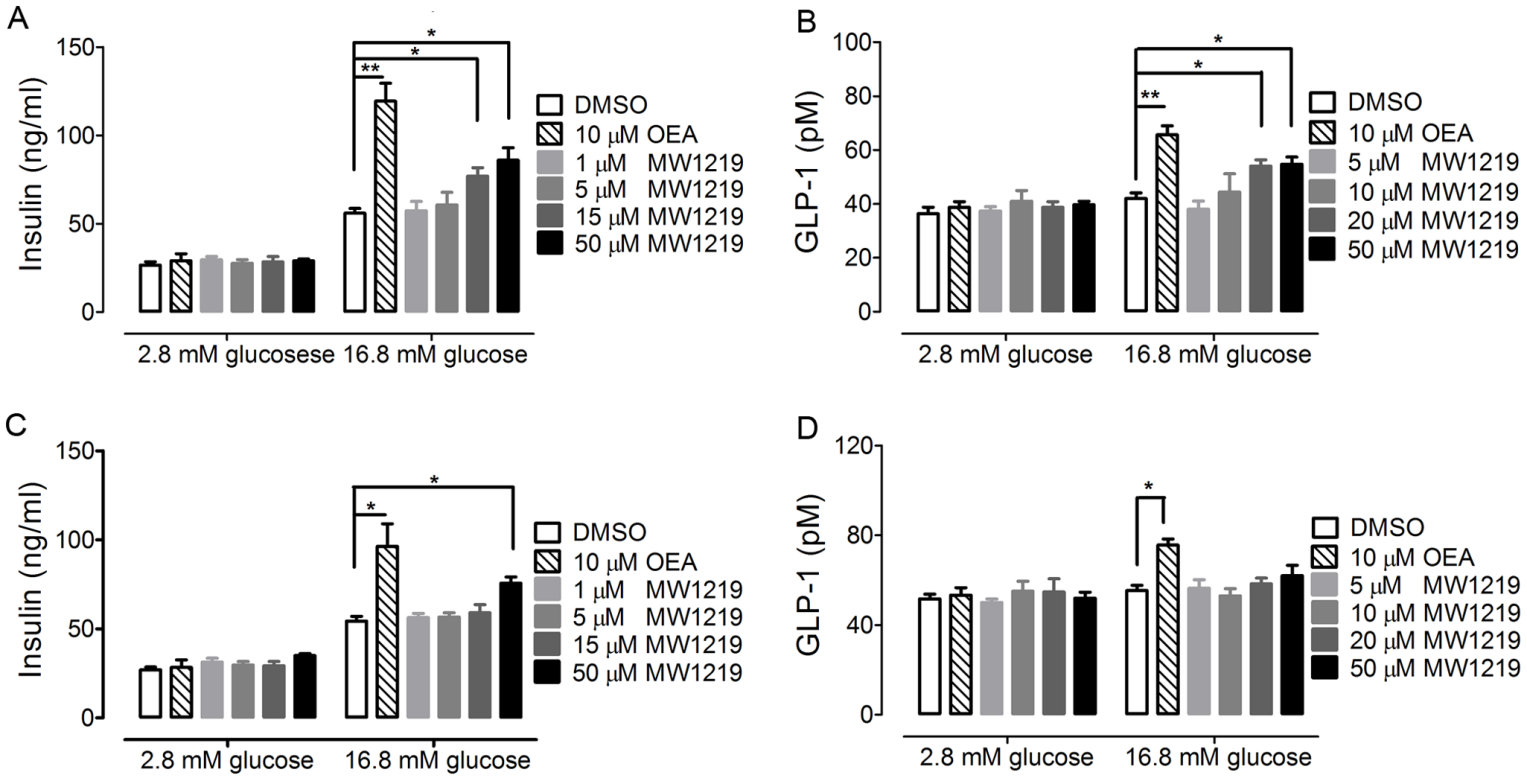
Effects of MW1219 on insulin and GLP-1 secretion ***in vitro*** **.** The effect of MW1219 to stimulate insulin secretion in MIN6 cells was studied in 2.8 mM and 16.8 mM glucose (A). Influence of GPR119 knockdown on MW1219-induced insulin secretion (C). Effect of MW1219 on GLP-1 release in GLUTag cells (B). After siRNA treatment, the effect of MW1219 on GLP-1 release was determined with GLUTag cells (D). All data points were representatives of three independent experiments, determined in triplicate. *P<0.05 and **P<0.01 denote a statistically significant difference between vehicle *vs.* compound-treated cells using One-Way ANOVA test.

To confirm the direct effects of MW1219 on pancreatic β-cells, we examined glucose-stimulated insulin secretion by mouse pancreatic MIN6 insulinoma cells which endogenously express GPR119 [16]. Two batches of MIN6 cells were exposed to 2.8 mM and 16.8 mM glucose medium, respectively. MIN6 cells exposed to 16.8 mM glucose increased insulin secretion when treated with 1 µM, 5 µM, 15 µM or 50 µM MW1219 or 10 µM OEA. Compared with DMSO control, MW1219 at 15 µM and 50 µM, as well as 10 µM OEA elevated insulin significantly (P<0.05 for MW1219, P<0.01 for OEA). In contrast, MW1219 had no effect on insulin release in MIN6 cells under low glucose (2.8 mM) conditions (Figure 3A).

To determine whether MW1219 could stimulate GLP-1 release, we measured the activity of MW1219 in GLUTag enteroendocrine cells expressing proglucagon gene and secreting GLP-1 in a regulated manner [17]. In high glucose conditions, the content of GLP-1 released to supernatant by GLUTag cells was elevated dose-dependently. Neither MW1219 nor OEA exhibited any effect in low glucose medium (Figure 3B).

We next studied if the insulinotropic effect of MW1219 described above is GPR119 dependent. Specific siRNA was employed to knockdown GPR119 in MIN6 cells and quantitative RT-PCR analysis demonstrated that the expression level of GPR119 was less than 70% of normal MIN6 cells (data not shown) accompanied by diminished effect of MW1219 on insulin secretion. The action of 15 µM MW1219 shown above was suppressed by siRNA and the significant difference between 15 µM MW1219 treatment and DMSO control disappeared (Figure 3C). A similar phenomenon was observed in GLUTag cells. The content of GLP-1 released by the cells treated with 20 µM and 50 µM MW1219 became indistinguishable from the medium control. Our results thus suggest that insulin and GLP-1 releases induced by MW1219 requires the presence of GPR119 (Figure 3D).

### Anti-diabetic Efficacy of Chronic MW1219 in db/db Mice

**Figure 4:**
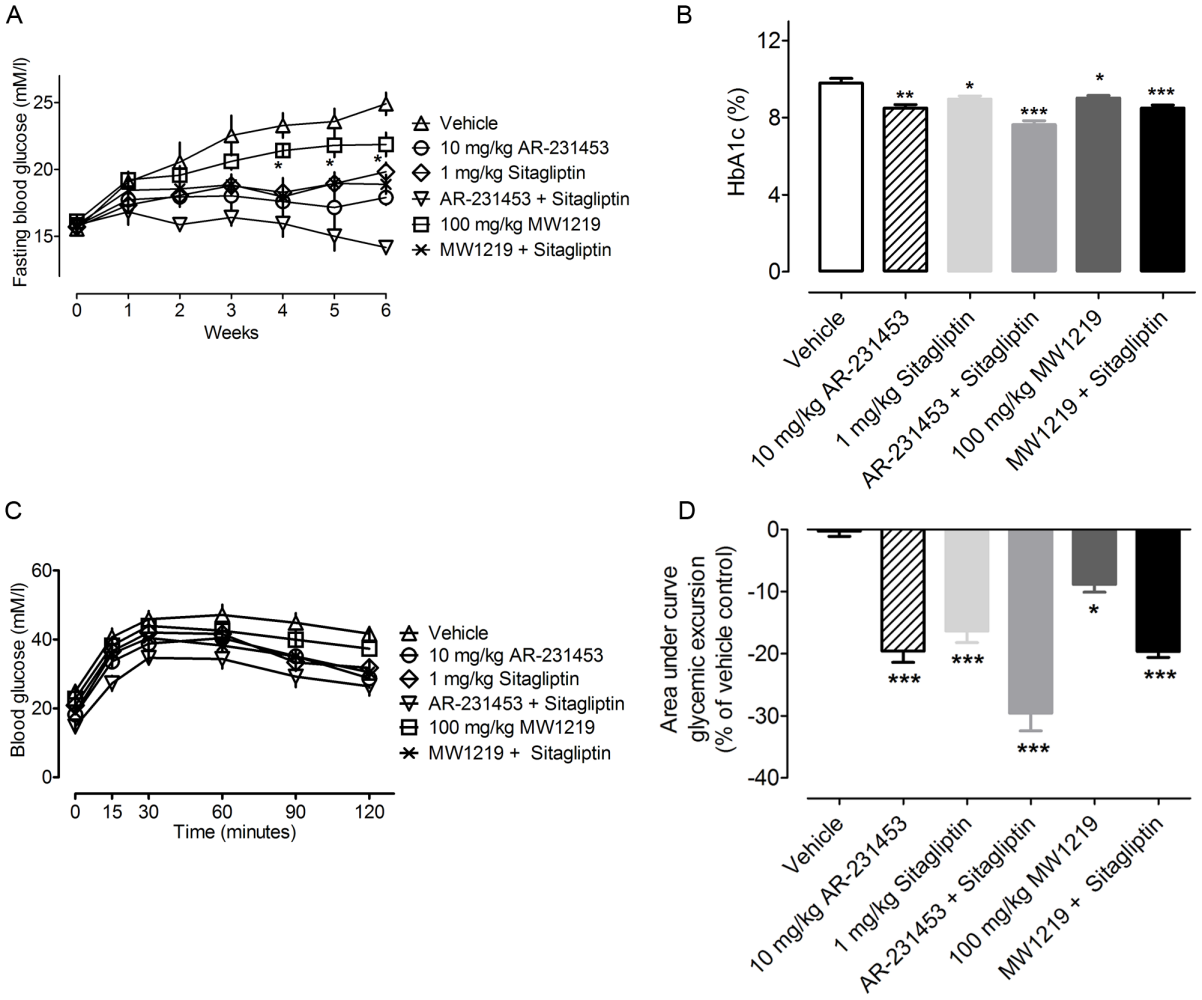
The effects of chronically administered MW1219 in ***db/db*** **mice.** (A) Time course of the effect on fasting blood glucose. After 6 weeks of treatment, (B) HbA1c, (C) oral glucose tolerance and (D) area-under-the-curve were quantified. Symbols are means ± SEM in all panels. *P<0.05, **P<0.01 *vs.* vehicle group, and ^#^P<0.05 *vs.* 1 mg/kg sitagliptin treatment group as determined with One-Way ANOVA test.

**Figure 5:**
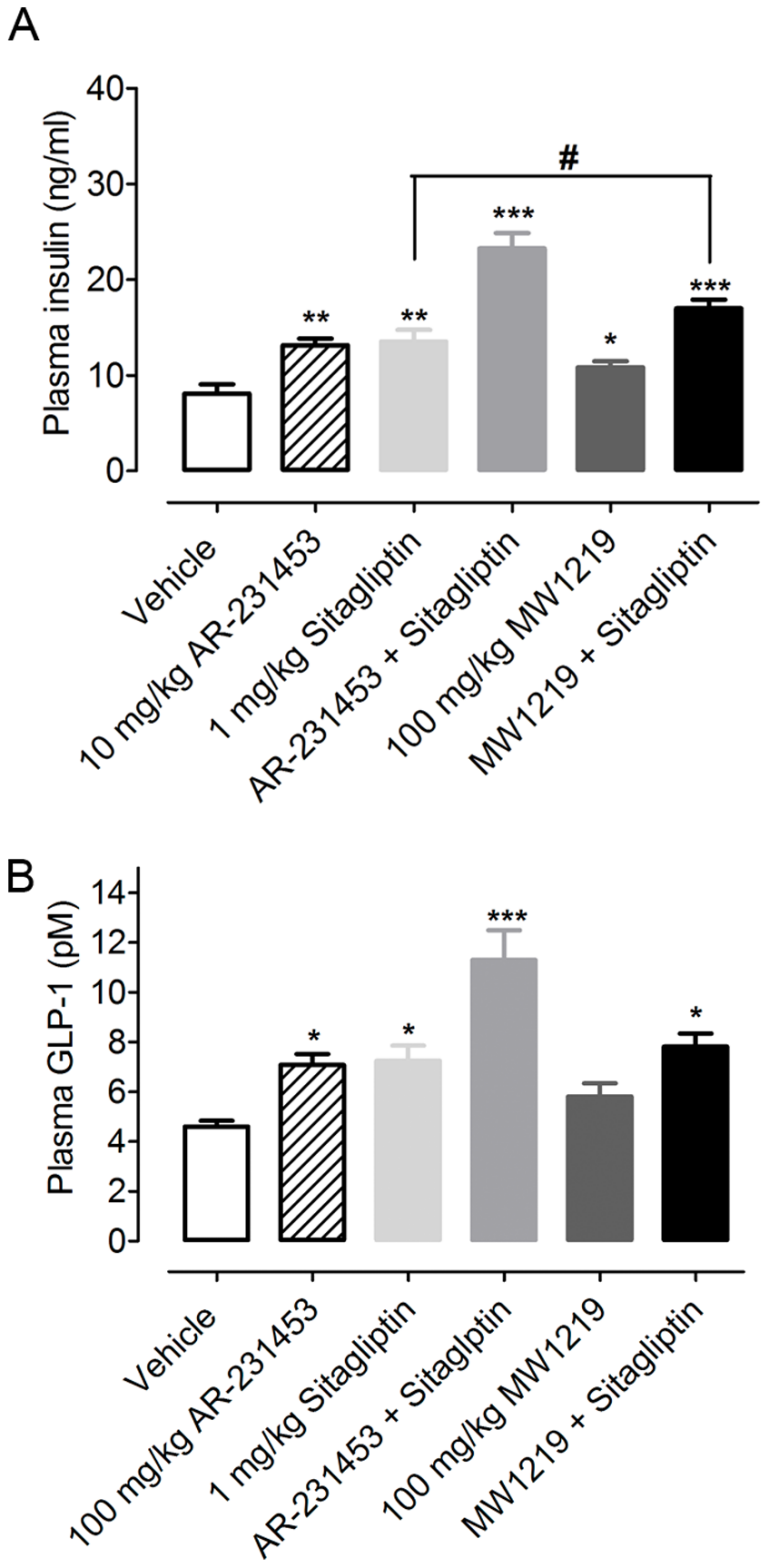
Activities of MW1219 on insulin and GLP-1 secretion in ***db/db*** **mice.** Following 6 weeks of oral treatment with MW1219, (A) insulin and (B) GLP-1 levels after an oral glucose bonus were determined. *P<0.05, **P<0.01 *vs.* vehicle group, and ^#^P<0.05 *vs.* sitagliptin group (1 mg/kg) as determined with One-Way ANOVA test.

To study potential glucose control property of MW1219 *in vivo*, male *db/db* mice were orally treated with various agents for 6 weeks. Food intake and body weight examined every other day during the treatment period did not show significant difference between six groups (Figure S1). After cessation of therapy, fasting blood glucose levels in all treatment groups markedly decreased compared with the control. Reduction by 16% and 29% was achieved following 100 mg/kg MW1219 and combination of MW1219 and 1 mg/kg sitagliptin administration, respectively. The level in the latter was significantly lower than that of mice received sitagliptin alone (Figure 4A). Through 6 weeks of treatment, HbA1c also decreased: compared with the control, there was a 0.67% decrease in animals treated with 100 mg/kg MW1219 (P<0.05); no difference was observed in 1 mg/kg sitagliptin and combination of MW1219 and sitagliptin treated groups (Figure 4B). Glucose tolerance tests demonstrate that 8.6% and 18.6% inhibition of glycemic excursion was realized in mice treated with either 100 mg/kg MW1219 or a combination of 100 mg/kg MW1219 and 1 mg/kg sitagliptin compared to the vehicle-treated control (P<0.05 and P<0.001, respectively; Figures 4C and 4D). The sensitivity of pancreas to glucose challenge was also improved following MW1219 treatment as insulin response to a glucose bolus was notably more pronounced than that of controls (Figure 5A). However, circulating GLP-1 levels after 100 mg/kg MW1219 administration was not elevated (Figure 5B).

## Discussion

GPR119 is coupled to Gα_s_ protein and activation of which can induce increases of cAMP. The cAMP accumulation and reporter gene assays described here are two functional methods mainly applied to identify and characterize hits in a HTS setting. Each assay has its own advantages and limitations [18]. Reporter gene assay has a number of amplification steps and this feature requires longer incubation time. It can increase the potential to identify both partial and full agonists which affords us to use it in HTS of GPR119 modulators including PAMs. After primary screening and subsequent confirmation, 248 hits were found (data not shown). MW1219 was selected based on its novel structure for further characterization.

MW1219 was capable of stimulating luciferase expression and cAMP accumulation in a concentration-dependent manner, but potency and efficacy varied between the two assay systems (Figures 2B to 2E). This discrepancy may be caused by different conditions and features of these two assays. For example, compound treatment time in the reporter gene assay (42 h) was much longer than that of the cAMP accumulation assay (30 min), and small changes in cAMP levels could be augmented by luciferase reaction in the reporter gene assay. Therefore, it appears that potency and efficacy measured by the reporter gene assay are generally more attractive. Nonetheless, this does not alter the fact that both assays identified MW1219 as a specific agonist for GPR119.

Compared with AR231453 and OEA, the molecular mass of MW1219 is smaller with a simple chemical structure. We studied its allosteric potential to AR231453 and OEA on GPR119. Our results show that MW1219 could increase their basal signals but not potencies to the receptor (Figures 2F and 2G). This phenomenon was also seen in the allosteric activity studies between AR231453 and OEA. It seems that MW1219 binds to the same site as AR231453 and OEA, and hence, is a pure GPR119 agonist.

Once GPR119 is activated, it will enhance the glucose-stimulated insulin release in pancreatic β cells. We thus studied this effect in MIN6 cells and the result showed that the compound was only effective in high glucose (16.8 mM) medium. After siRNA treatment, this action of MW1219 was suppressed, a property consistent with other GPR119 agonists reported previously [19]. This compound was also evaluated in GLUTag cells and the result was similar to that seen in MIN6 cells: MW1219 could only enhance GLP-1 release in 16.8 mM glucose medium. These data suggest that MW1219, when used *in vivo*, may devoid hypoglycemia, a complication commonly associated with insulin therapy.

Encouraged by our pilot dose-response studies *in vivo* in which MW1219 at 100 mg/kg improved both fasting plasma glucose levels and glucose tolerance (data not shown), we further examined the anti-diabetic effects of MW1219, either alone or in combination with sitagliptin in *db/db* mice as presented in this paper. Clearly, subchronic treatment of diabetic mice with MW1219 led to reduction in HbA1c and fasting glucose levels accompanied by improved glucose tolerance and insulin sensitivity. Additive effect was observed when it was co-administered with sitagliptin, pointing to a potential as a drug lead for further development.

Obesity is one of the most important factors in the development of insulin resistance. Associated with obesity, metabolic disorders including hyperinsulinemia, impaired glucose tolerance and dyslipidemia are often noted, which increase the risk for type 2 diabetes. GPR119 can stimulate GLP-1 secretion resulting in suppression of food intake [20] and gastric emptying [21]. Some agonists of GPR119, such as OEA, are also known to reduce food intake [9], [22]. However, we observed neither food intake inhibition nor weight loss following 6-week MW1219 treatment (Figure S1). According to the literature, AR-231453 could reduce food intake only at very high doses. Sitagliptin at 1 mg/kg raised plasma GLP-1 levels but failed to suppress food intake and decrease body weight. Combination of AR-231453 with sitagliptin also did not induce noticeable effects on feeding and weight gain. Thus, our observation on these two metabolic parameters after MW1219 intervention is in line with previous findings.

Finally, we also evaluated the ability of MW1219, either alone or in combination with sitagliptin, to stimulate the release of insulin and GLP-1 in *db/db* mice. After an oral glucose bolus, levels of insulin and GLP-1 were elevated at 20 min and 2 min, respectively [7], [10]. In our hands, we found that MW1219 at 100 mg/kg was only able to induce the secretion of insulin (Figure 5A) but not GLP-1 (Figure 5B). This may be explained by the fact that MW1219 is a weak GPR119 agonist and it is generally accepted that GLP-1 is hard to measure due to its short half-life [23].

In summary, we have identified a novel small molecule (MW1219) capable of activating GPR119 and exerting beneficial metabolic effects *in vitro* and *in vivo*. Like other GPR119 agonists reported so far, MW1219 does not show any allostreric activities and comparison with AR231453, the agonist activity of MW1219 is rather weak. Obviously, this would not prevent it from becoming a scaffold for structural modification and optimization through medicinal chemistry efforts.

## Supporting Information

Figure S1 **Effects of chronic MW1219 treatment on food intake and body weight in** ***db/db*** **mice.** (A) Food intake in *db/db* mice treated with different regimens; (B) Body weight after 6 weeks of treatment. Data are shown as means ± SEM (n = 10). *P<0.05, **P<0.01 *vs.* vehicle group as determined with One-Way ANOVA test.
(TIF)

Table S1 Summary of allosteric modulation of AR-231453 on OEA in the reporter gene and cAMP accumulation assays.
(DOC)

Table S2 Summary of allosteric modulation of OEA on AR-231453 in the reporter gene and cAMP accumulation assays.
(DOC)

Table S3 Summary of allosteric modulation of MW1219 on OEA in the reporter gene and cAMP accumulation assays.
(DOC)
