# Supplementary figures and images for: High-Throughput Screening for GPR119 Modulators Identifies a Novel Compound with Anti-Diabetic Efficacy in db/db Mice
Source: PLoS One. 2013 May 21;8(5):e63861. doi: 10.1371/journal.pone.0063861 (PMC3660563; doi:10.1371/journal.pone.0063861)

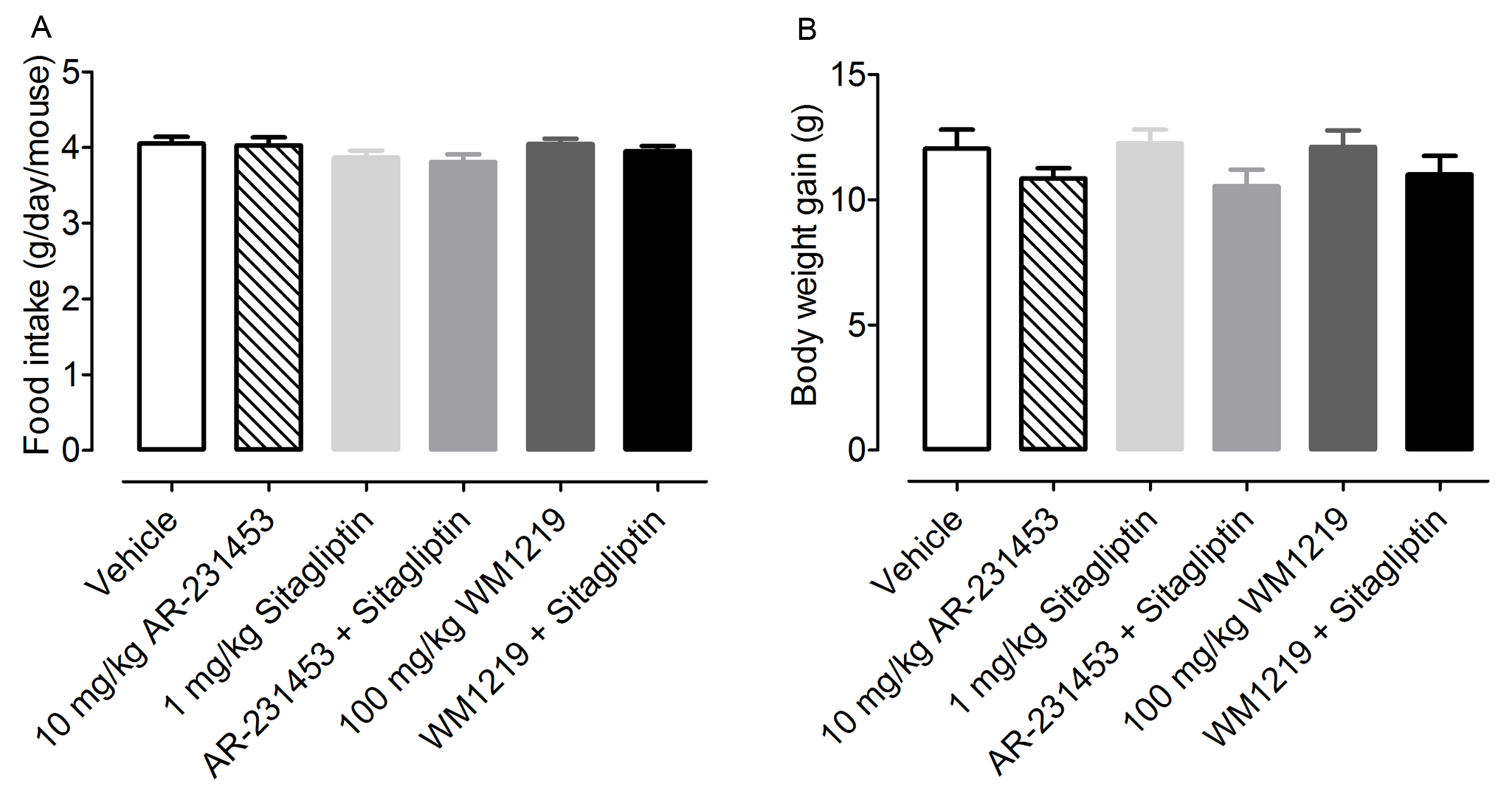

Supplement: Figure S1 — Effects of chronic MW1219 treatment on food intake and body weight in db/db mice. (A) Food intake in db/db mice treated with different regimens; (B) Body weight after 6 weeks of treatment. Data are shown as means ± SEM (n = 10). *P<0.05, **P<0.01 vs. vehicle group as determined with One-Way ANOVA test. (TIF) [file pone.0063861.s001.tif]
