# Supplementary material for: High-Throughput Screening for GPR119 Modulators Identifies a Novel Compound with Anti-Diabetic Efficacy in db/db Mice
Source: PLoS One. 2013 May 21;8(5):e63861. doi: 10.1371/journal.pone.0063861 (PMC3660563; doi:10.1371/journal.pone.0063861)
Supplement: Table S1 — Summary of allosteric modulation of AR-231453 on OEA in the reporter gene and cAMP accumulation assays. (DOC) [file pone.0063861.s002.doc]

Table S1. Summary of allosteric modulation of AR-231453 on OEA in the reporter gene and cAMP accumulation assays.

| **Reporter gene assay** | | **cAMP accumulation assay** | |
| --- | --- | --- | --- |
| **AR-231453 (nM)** | **EC_50_ of OEA (μM)** | **AR-231453 (nM)** | **EC_50_ of OEA (μM)** |
| 1 | 2.65±0.44 | 1 | 1.61±0.16 |
| 0.2 | 2.38±0.51 | 0.2 | 1.73±0.19 |
| 0.04 | 2.91±0.62 | 0.04 | 1.54±0.27 |
| 0.008 | 2.55±0.37 | 0.008 | 1.67±0.31 |
| 0 | 2.81±0.21 | 0 | 1.54±0.28 |
