# Supplementary material for: High-Throughput Screening for GPR119 Modulators Identifies a Novel Compound with Anti-Diabetic Efficacy in db/db Mice
Source: PLoS One. 2013 May 21;8(5):e63861. doi: 10.1371/journal.pone.0063861 (PMC3660563; doi:10.1371/journal.pone.0063861)
Supplement: Table S2 — Summary of allosteric modulation of OEA on AR-231453 in the reporter gene and cAMP accumulation assays. (DOC) [file pone.0063861.s003.doc]

Table S2. Summary of allosteric modulation of OEA on AR-231453 in the reporter gene and cAMP accumulation assays.

| **Reporter gene assay** | | **cAMP accumulation assay** | |
| --- | --- | --- | --- |
| **OEA (μM)** | **EC_50_ of AR-231453 (nM)** | **OEA (μM)** | **EC_50_ of AR-231453 (nM)** |
| 50 | 1.29±0.19 | 10 | 1.26±0.12 |
| 15 | 1.05±0.03 | 3 | 0.94±0.05 |
| 5 | 1.00±0.09 | 1 | 1.03±0.06 |
| 1.5 | 0.97±0.12 | 0.3 | 0.93±0.05 |
| 0.5 | 1.01± 0.23 | 0.1 | 1.06±0.13 |
| 0 | 1.05± 0.11 | 0 | 1.19±0.16 |
