# Supplementary material for: High-Throughput Screening for GPR119 Modulators Identifies a Novel Compound with Anti-Diabetic Efficacy in db/db Mice
Source: PLoS One. 2013 May 21;8(5):e63861. doi: 10.1371/journal.pone.0063861 (PMC3660563; doi:10.1371/journal.pone.0063861)
Supplement: Table S3 — Summary of allosteric modulation of MW1219 on OEA in the reporter gene and cAMP accumulation assays. (DOC) [file pone.0063861.s004.doc]

Table S3. Summary of allosteric modulation of MW1219 on OEA in the reporter gene and cAMP accumulation assays.

| **Reporter gene assay** | | **cAMP accumulation assay** | |
| --- | --- | --- | --- |
| **MW1219 (μM)** | **EC_50_ of OEA (μM)** | **MW1219 (μM)** | **EC_50_ of OEA (μM)** |
| 10 | 2.56±0.47 | 10 | 1.41±0.11 |
| 1 | 3.09±0.68 | 1 | 1.83±0.24 |
| 0 | 2.89±0.61 | 0 | 1.69±0.17 |
